# Implementation of an ISBT 128-Compatible Medical Record System to Facilitate Traceability of Stem Cell Products

**DOI:** 10.4274/tjh.2017.0081

**Published:** 2017-08-02

**Authors:** Can Boğa, Erkan Maytalman, Çiğdem Gereklioğlu, Süheyl Asma, Fatih Kandemir, Pelin Aytan, Aslı Korur, Mahmut Yeral, İlknur Kozanoğlu, Hakan Özdoğu

**Affiliations:** 1 Başkent University Faculty of Medicine, Department of Hematology, Ankara, Turkey; 2 Başkent University Faculty of Medicine, Department of Family Medicine, Ankara, Turkey; 3 Başkent University Faculty of Medicine, Department of Physiology, Ankara, Turkey

**Keywords:** Hematopoietic stem and progenitor cells, Stem cell mobilization, Apheresis, ISBT 128, JACIE, Labeling, Traceability

## To The Editor,

The Foundation for the Accreditation of Cellular Therapy and the Joint Accreditation Committee-ISCT & EBMT (FACT-JACIE) demands the tracking of stem cell products, beginning from the harvesting of the product from the patient or donor and continuing until the time of use in stem cell transplantation centers [1]. Relevant follow-up stages for stem cell product bags and vials include harvesting, portioning, transportation, acceptance at the cell processing unit, processing, storage, recycling, release, acceptance at the clinical unit, infusion, and recall at the cellular processing unit [1,2,3,4]. Information Standard for Blood and Transplant (ISBT) 128 provides unique identification and traceability of stem cell products using an international coding system [5]. Although this system is required to be implemented in accredited centers, many centers could not yet begin using this system. Sufficient data are not available in the literature about whether this system has facilitated workflow or not. This study was planned in order to investigate the feasibility of the ISBT 128 coding system.

This is a single-center, cross-sectional, and prospective study conducted at a JACIE-accredited center between January 2012 and December 2016. Cellular therapy production codes beginning with ‘S’ were checked against the International Council for Commonality in Blood Banking Automation registry and unique identifiers for patient, donor, and stem cell products were produced. The class, modifier, and additives for the product were defined using the terminology table [6]. ISBT-compatible software (Turunç v.0.2, Teknik Media, Adana, Turkey) was used as the medical recording system. The function and the continuity of the system were evaluated every 15 days. Time to reach the data of the stem cell product at a certain time between harvesting and infusion/disposal and system implementation problems were evaluated. For this purpose, cell product characteristics were evaluated at every stage for 20 randomly selected products.

**Figure 1:**
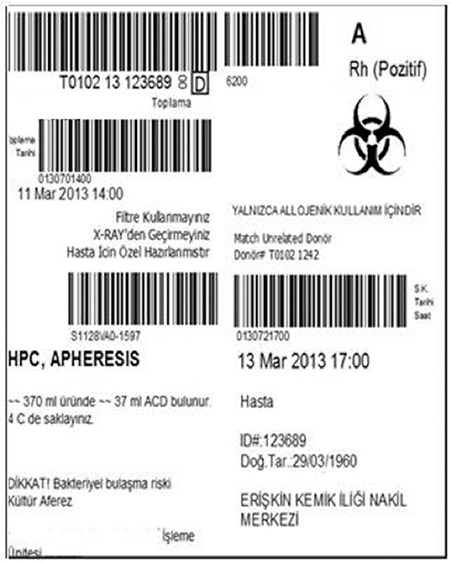
Bar code denotes donation identification number (upper left), blood group (upper right), collection (or production) date and time (middle), product code (lower left), and expiration date and time (lower right). The biohazard mark was placed correctly on the labels of products that were detected to pose infection risks.

A total of 2703 records belonging to 467 patients/donors were analyzed. The distribution of record numbers according to stages of the cellular product’s journey were 712 for cell collection, 1460 for cell processing, 2 for recall of the product, and 561 for disposal of the product. A sample of a final allogeneic label is shown in Figure 1. The biohazard mark and a written warning regarding infectious agents were placed correctly on the labels of the infection-positive products. The time to reach data of cell content, portion number, storage location of bags, and storage location of vials were 6.1±1.1 s, 5.3±1 s, 6.4±0.9 s, and 6.4±0.9 s, respectively. No deviation from quarantine procedure was identified. Only three label production errors were detected (0.097%). No torn labels were produced.

The coding system was seen to facilitate workflow by enabling communication between transplant units. Labels that were structured in accordance with ISBT 128 could be produced at appropriate stages [7,8]. Almost no mix-up error being found in our study is a striking result to ensure the safety and reliability of the system.

The ISBT 128 system was found to be effective for traceability of stem cell products during their journey from harvesting to infusion/disposal and it facilitates the workflow in clinical practice in transplant and cellular therapy centers.
